# 7th International Workshop on SOX Transcription Factors

**DOI:** 10.1242/bio.062466

**Published:** 2026-08-10

**Authors:** Aya Uchida

**Affiliations:** Research and Development, The Jackson Laboratory Japan, Ishioka 315-0138, Japan

**Keywords:** SOX transcription factor, Sex determination, Disorders of sex development, Organoids, Gene therapy, Comparative and evolutionary biology

## Abstract

Marking the 20th anniversary of a well-established meeting series focused on the biological and clinical importance of *SOX* genes as regulators of cell fate, the 7th International Workshop on SOX Transcription Factors held from 8−11 September 2025 at the Prince Resort MICE Karuizawa in Nagano, Japan, brought together 83 participants from 17 countries. The meeting showcased the foundations of *SOX* gene research and recent technological and conceptual breakthroughs across diverse model systems and human research, illustrating the field's evolution and emerging directions for future research. Reflecting the breadth of SOX biology – from early embryonic development and sex determination to pathogenesis and sex testing policies in sports – this Meeting Review highlights the key scientific advances and messages presented at the meeting to encourage and inspire researchers in the field of SOX biology.

## Teaser

**Summary:** This Meeting Review highlights key advances in SOX biology presented at the meeting, spanning the roles of *SOX* genes in development and disease, emerging model systems, and ongoing translation into diagnosis and therapeutics.

## INTRODUCTION

The sex-determining region Y (SRY)-related high-mobility group (HMG) box (SOX) family consists of genes characterized by a highly conserved HMG box domain that shares over 60% sequence homology with the founding family member, SRY (Gubbay et al., 1990; Koopman et al., 1991; Sinclair et al., 1990). SOX transcription factors serve as key regulators of cell fate determination and differentiation across a wide range of organ systems, directing biological processes from pluripotent stem cells to terminally differentiated cells. Disruption of *SOX* gene function is associated with a wide spectrum of human disorders, including sex reversal, congenital malformations, neurodevelopmental syndromes, pulmonary arterial hypertension, and various cancers, highlighting their clinical relevance and therapeutic potential.

The SOX conference series has long provided a platform to unite researchers from diverse disciplines with a shared interest in SOX biology, fostering collaboration, supporting future research, and encouraging broad participation in advancing the field. In celebration of its 20th anniversary, the 7th International Workshop on SOX Transcription Factors was organized by founding members of the series, including Masami Kanai-Azuma and Hiroshi Asahara (Institute of Science Tokyo, Japan), Yoshiakira Kanai (The University of Tokyo, Japan), and co-organizers Martin Cheung and Ralf Jauch (The University of Hong Kong, China); their leadership brought together pioneering researchers and emerging investigators.

From the early discovery of SRY to the present day, our foundational understanding of *SOX* genes has advanced substantially, extending beyond classical gene knockout studies into epigenetic regulation and functional genomics. However, several key aspects remain to be addressed. These include translating findings from diverse animal models across species, as well as further elucidating molecular mechanisms of sex determination beyond the presence of *SRY*. Because direct *in vivo* experimental manipulation remains limited in many settings, there is a strong need to develop *in vitro* systems that more faithfully recapitulate tissue organization, physiology, and disease states. A deeper understanding of disease mechanisms, together with the development of SOX-related genes as tools for diagnosis and therapeutic intervention remains an important goal, along with the need to further translate these findings into clinical and societal applications. During this meeting, leading figures from diverse fields presented their ongoing efforts to address these challenges. This review aims to highlight the key advancements and perspectives shared during the meeting. Although the conference was a closed meeting focusing on unpublished data, some findings presented at the meeting have subsequently become publicly available through publications and reports (Kanai-Azuma et al., 2026), allowing discussion of these findings in this review.

## Cross-species translation of SOX biology

This meeting was unique in bringing together researchers working across diverse animal models, *in vitro* systems, and human studies, providing a valuable opportunity to integrate collective insights in SOX biology. Participants highlighted the enduring value of fundamental *in vivo* research using animal models, while also emphasizing the need for cross-species translation of insights obtained from diverse model systems. As one example, Naoko Irie (Keio University, Japan) discussed species-specific differences in primordial germ cell (PGC) specification. While SOX17 is essential for human PGC specification (Irie et al., 2015), it is dispensable in mice (Kanai-Azuma et al., 2002), highlighting a striking divergence in developmental regulatory mechanisms. She further examined the interplay between SOX17 and other SOX family members in PGCs, suggesting species-specific regulatory compensation and differential utilization of *SOX* genes during evolution. In contrast, Ralf Jauch (The University of Hong Kong, China) highlighted the deep evolutionary conservation of *SOX* genes, emphasizing that SOX transcription factors predate the emergence of multicellular animals. He demonstrated that unicellular SOX proteins bind DNA in a manner similar to mammalian SOX2 and can contribute to the induction of pluripotent stem cell states. Strikingly, SOX proteins from choanoflagellates can induce pluripotency in mouse cells, while engineered SOX17 can functionally substitute for SOX2 during human cell reprogramming (Hu et al., 2026). These findings highlight remarkable functional conservation across species as well as unexpected interchangeability among distinct SOX family members within the same species. Functional interchangeability among SOX factors was further demonstrated within *SoxB* group by Steve Russell (University of Cambridge, UK), who showed that the mouse *SOX2* coding sequence can fully substitute for *SoxNeuro* function in *Drosophila*. These findings underscore the remarkable evolutionary conservation of SOX-mediated regulatory mechanisms, while also revealing important species-specific differences in how individual *SOX* genes are deployed. Dissecting the balance between conserved functions and lineage-specific utilization of SOX factors remains an important area for future investigation.

## Diverse sex determination mechanism in mammals

Although the identification of SRY as a master regulator of male gonadal development laid the foundation for SOX gene research, the meeting reflected the field's growing recognition of the complex landscape of sex determination, spanning context-dependent regulation of *SOX* genes, mechanisms beyond the canonical SRY-dependent model, and the remarkable plasticity of gonadal fate after birth. Makoto Tachibana (The University of Osaka, Japan) presented a novel perspective on how environmental context can shape SRY regulation, by demonstrating that ferrous iron (Fe^2+^) metabolism regulates the epigenetic activation of *Sry* through KDM3A, an H3K9 demethylase (Okashita et al., 2025). His findings revealed an unexpected link between maternal dietary iron and fetal male sex determination, highlighting maternal nutrition as a previously unrecognized regulator of this process. Shogo Matoba (Riken BioResoruce Research Center, Japan) further discussed how Y-linked genes beyond *Sry* contribute to sexual dimorphism and presented an approach to quantify the contribution of individual sex-linked genes across 53 sexually dimorphic traits (Tanaka et al., 2026), highlighting additional layers of regulation beyond the canonical SRY-dependent mechanism of sex determination. Robin Lovell-Badge (The Francis Crick Institute, UK) and Shizuka Kirino (Institute of Science of Tokyo, Japan) discussed the intriguing phenomenon of mouse postnatal gonadal sex reversal driven by the transdifferentiation of ovarian granulosa cells into Sertoli-like cells following the loss of *Foxl2*. Although this phenomenon has been known for some time (Uhlenhaut et al., 2009), the underlying mechanisms remained unclear. They addressed this longstanding mystery using advanced ChIP-seq and scRNA-seq analyses, uncovering the interplay between FOXL2 and SOX9 as well as unique characteristics of the transdifferentiated cells, respectively (Stévant et al., 2025). This rare example of fate conversion in a differentiated organ highlights remarkable plasticity in postnatal gonads, although its biological significance in mammals remains unclear and needs to be explored further.

## Clinical and societal implications of sex determination research

Insights gained from SOX research in sex determination are also beginning to translate into clinical practice and broader societal applications, particularly in the context of disorders of sex development (DSD). Drawing on clinical data from more than 1000 patients with DSD, Katie Ayers (Murdoch Children's Research Institute, Australia) highlighted how genetic variants, copy number alterations, and changes in the expression or function of *SOX* genes contribute to disease pathogenesis. As one example, she showed that duplication or deletion of a core enhancer regulating SOX9 can result in XX male sex reversal and XY female sex reversal, respectively (Croft et al., 2018). Beyond its relevance to DSD, SOX biology also carries important societal implications. Andrew Sinclair (Murdoch Children's Research Institute, Australia) discussed how advances in the field could help inform ongoing controversies surrounding the use of SRY gene testing in women's sports. He noted that SRY testing only detects the presence of the *SRY* gene and does not reflect its functional status or the body's response to testosterone, thereby failing to identify individuals with androgen insensitivity syndrome or other disorders of sex development (Bowman-Smart et al., 2024). These limitations highlight the need for more nuanced scientific and ethical frameworks for evaluating eligibility to compete in women's sports.

## Emerging *in vitro* models for developmental research

Building on insights gained from basic SOX research, we are now entering an era in which stem cell–based systems, including organoids and blastoids, enable increasingly sophisticated *in vitro* modelling. Katsuhiko Hayashi (The University of Osaka, Japan) presented his group's recent breakthrough in testicular organoid generation (Yoshino et al., 2026; Sato et al., 2026), building on his pioneering work in pluripotent stem cells-derived primordial germ cell-like cells (PGCLCs) and *in vitro* oogenesis (Saitou and Hayashi, 2021), including oocyte generation from XY ES cells (Murakami et al., 2023). Generation of testicular organoids that faithfully recapitulate testicular anatomy with properly organized seminiferous tubules has long remained a major challenge. Hayashi and colleagues overcame this limitation by refining culture conditions to generate testicular organoids from mouse XY ES cells that preserve seminiferous tubule architecture. Using this system, his group demonstrated that spermatogonia generated within the organoids could further develop into sperm and produce viable offspring following transplantation into recipient mouse testes. Extending this platform, he discussed reconstructing the process of sex determination, from bipotential gonads to testicular somatic cell-like cell (TesLC) differentiation, using both germ cells and gonadal somatic cells derived from embryonic stem cells. He further demonstrated how experimental recombination of these cells could be used to model gonadal sex reversal *in vitro*. Although achieving complete *in vitro* spermatogenesis remains challenging due to the inability to fully recapitulate meiosis and generate haploid cells, this work represents a major technical milestone for studying gonadal sex determination, as well as testicular biology *in vitro*. These advances are further complemented by Katie Ayers's presentation on recent progress in human iPSC-derived testis-like organoids that can model multiple gonadal cell lineages and DSD-related developmental processes (Pachernegg et al., 2026). Beyond gonadal organoid systems (Saitou and Hayashi, 2021), emerging next-generation stem cell-based models are also expanding the scope of developmental biology research. As a cutting-edge model for studying human embryogenesis and implantation mechanism, Ayaka Yanagida (The University of Tokyo, Japan) and Hiroo Sasaki (The University of Tokyo, Japan) presented their work using human and mouse blastoids (Yanagida et al., 2021) to dissect conserved and divergent developmental mechanisms across species. Such advanced *in vitro* models provide powerful platforms for studying gametogenesis and embryogenesis in greater depth, enabling innovative experimental approaches. However, their application to human biology raises important ethical considerations, and regional differences in regulatory frameworks may influence the pace and scope of research. In this context, international collaboration will be essential to advance the field responsibly.

## SOX in disease mechanisms and therapeutic target candidates

Growing evidence indicates that *SOX* genes play central roles in disease pathogenesis beyond development, revealing new opportunities for therapeutic target discovery. Sanjeev Kumar (Cedars-Sinai Medical Center, USA) demonstrated the essential role of SOX9 in mammalian nephron development and repair, showing that its temporal regulation critically influences tissue recovery following acute kidney injury (AKI). Prolonged SOX9 expression promotes fibrosis, whereas its timely downregulation facilitates regeneration (Callemeyn et al., 2024). Similarly, Joerg Heineke (Heidelberg University, Germany) identified endothelial SOX9 as a driver of endothelial-to-mesenchymal transition and fibroblast activation in organ fibrosis. He further demonstrated epicardial SOX9 deletion reduces inflammation and skin fibrosis in mice (Trogisch et al., 2024), underscoring the importance of SOX9 as a potential therapeutic target in fibrotic disease. In addition, Jun-Bean Park (Seoul National University, Korea) and Injune Kim (Korea Advanced Institute of Science and Technology, Korea) highlighted how disruption of SOX17 results in pulmonary arterial hypertension (PAH) (Park et al., 2022), while Christopher James Rhodes (Imperial College London, UK) demonstrated the novel therapeutic compounds targeting *Sox17* signaling could rescue transcriptomic alterations induced by SOX17 loss, emphasizing its emerging potential as a therapeutic target (Walters et al., 2023).

## Development of SOX-targeted therapeutics

Building on decades of basic research on *SOX* genes, recent advances in gene therapy are accelerating the translation of SOX biology into therapeutic strategies, creating new opportunities to treat disorders caused by SOX mutations or dysregulation that currently lack effective therapies. Véronique Lefebvre (Children's Hospital of Philadelphia, USA) presented a proof-of-concept study targeting Lamb-Shaffer syndrome, a neurodevelopmental disorder caused by genomic variants that inactivate one allele of *SOX5* (Zawerton et al., 2020). While the disorder can be diagnosed through genetic testing, no effective treatments currently exist. To address this unmet need, her group developed a SOX5-AAV vector and evaluated its therapeutic potential in mouse models and human iPSC-derived cortical organoids. Steven Pollard (The University of Edinburgh, UK) highlighted the therapeutic potential of targeting SOX2- and SOX9-driven gene networks in glioblastoma stem cells, presenting a synthetic super-enhancer (SSE)-driven AAV gene therapy approach in which engineered transcriptional switches selectively target aggressive glioblastoma cells. His recent preclinical data demonstrated complete tumor eradication and durable protection in aggressive glioma models (Koeber et al., 2026). This strategy may also have broader applications in diseases requiring precise, cell state-specific control of transgene expression. Taken together, these talks emphasized the diverse role of *SOX* genes in disease pathogenesis and underscored how advances in therapeutic development are bringing clinical applications increasingly within reach, highlighting the broad translational potential of SOX biology across multiple disease contexts.

## Encouraging network for future scientific advances

The meeting encouraged scientific exchange and collaboration across the SOX research community by bringing together scientists who might not otherwise intersect.

**Fig. 1.**
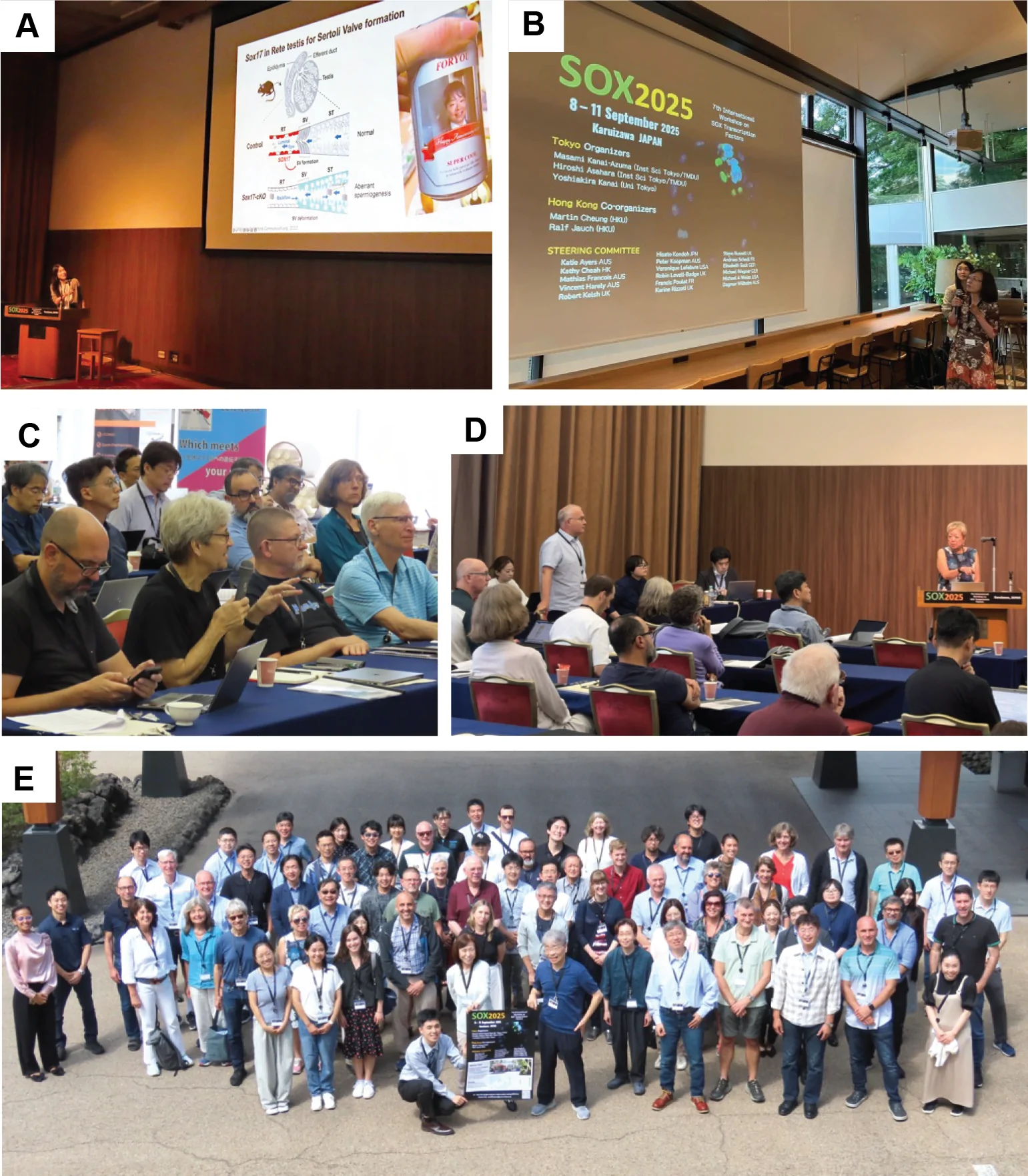
**Overview of the 7th International SOX Research Conference.** (A) The first author presenting during a scientific session. (B) Opening remark delivered at the reception. (C,D) Attendees during the conference sessions. (E) Group photograph of the attendees at the 7th International Workshop on SOX Transcription Factors held at Prince Resort MICE Karuizawa in Nagano, Japan. The photographs were provided by the organizers.

**Fig. 2.**
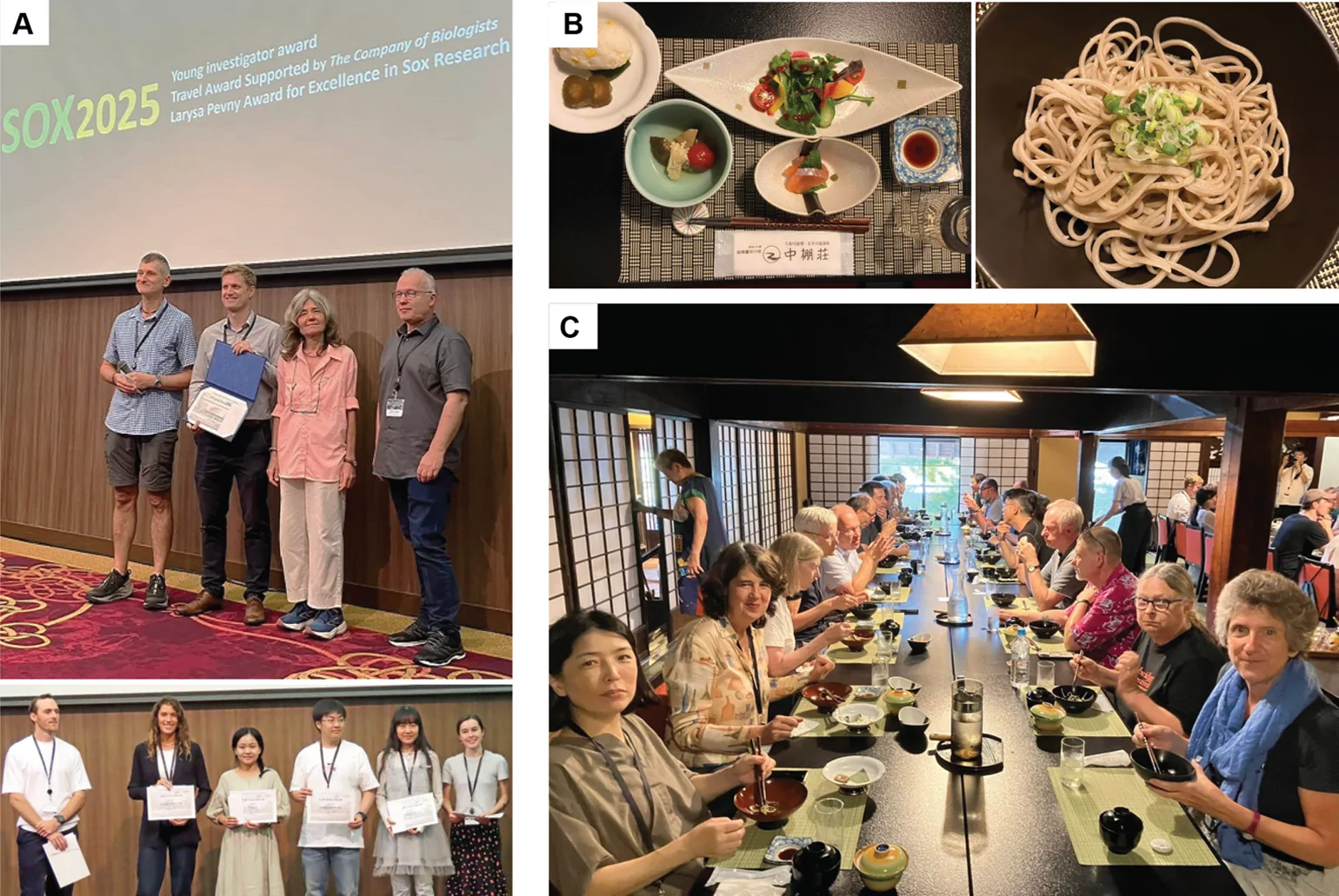
**Social events and award presentations at the conference.** (A) The Larysa Pevny Prize and Travel awards, supported by The Company of Biologists. (B,C) Lunch including soba noodles at the traditional Japanese restaurant Nakadanasou and participants during the conference-hosted excursion. All photographs were provided by the organizers.

The author attended this conference series for the first time to present previous and ongoing work on SOX biology in mouse spermatogenesis (Uchida et al., 2022) and trophoblast stem cells (Fig. 1A), experiencing firsthand the inclusive and welcoming atmosphere of the meeting. Reflecting this atmosphere, 66 of the 87 participants, ranging from faculty members to undergraduate students, presented their work through 44 talks and 22 posters. The meeting also placed strong emphasis on supporting early-career researchers. The Larysa Pevny Prize was awarded to two young principal investigators, Naoko Irie and Christopher James Rhodes, in recognition of their outstanding contributions to SOX research (Fig. 2A). Travel awards supported the participation of six early-career researchers who presented posters at the meeting (Fig. 2A). Furthermore, a conference-hosted excursion offered a valuable opportunity to strengthen professional and personal connections while enjoying soba noodles at the traditional Japanese restaurant called Nakadanasou and exploring Komoro Castle Ruins Park (Fig. 2B,C). Overall, the meeting fostered broader perspectives as well as professional networks and friendships that will collectively support scientific advances.

## Conclusions

Uniting researchers from diverse fields and perspectives around the unifying theme of SOX biology, the meeting highlighted ongoing efforts to understand SOX-mediated developmental and disease mechanisms across species and model systems, while advancing *in vitro* platforms and translational applications of findings in the field. The conference offered a valuable opportunity to gain early insight into emerging discoveries and technological advances while engaging in direct discussions with leading researchers in the field. Equally important, the meeting fostered scientific exchange and collaboration across disciplines and career stages, nurturing professional networks and future collaborations within SOX research community. The SOX conference will undoubtedly continue to nurture these opportunities, with the next meeting scheduled to be held in Hong Kong in 2028.
